# Short- and Long-Term Immunological Responses in Chronic HCV/HIV Co-Infected Compared to HCV Mono-Infected Patients after DAA Therapy

**DOI:** 10.3390/pathogens10111488

**Published:** 2021-11-15

**Authors:** Stefania Farcomeni, Sonia Moretti, Caterina Fimiani, Lucia Fontanelli Sulekova, Fenicia Vescio, Leonardo Sernicola, Maria T. Maggiorella, Anna Lisa Remoli, Orietta Picconi, Luciana Mosca, Rozenn Esvan, Elisa Biliotti, Massimo Ciccozzi, Marco Sgarbanti, Gloria Taliani, Alessandra Borsetti

**Affiliations:** 1 National HIV/AIDS Research Center, Istituto Superiore di Sanità, 00161 Rome, Italy; stefania.farcomeni@iss.it (S.F.); sonia.moretti@iss.it (S.M.); leonardo.sernicola@iss.it (L.S.); mariateresa.maggiorella@iss.it (M.T.M.); orietta.picconi@iss.it (O.P.); 2 Department of Public Health and Infectious Diseases, “Sapienza” University of Rome, 00161 Rome, Italy; c.fimiani@policlinicoumberto1.it (C.F.); rozenn.esvan@inmi.it (R.E.); 3 Department of Translation and Precision Medicine, “Sapienza” University of Rome, 00161 Rome, Italy; lucia.fontanellisulekova@uniroma1.it; 4 Department of Infectious Diseases, Istituto Superiore di Sanità, 00161 Rome, Italy; fenicia.vescio@iss.it (F.V.); aremoli@gmail.com (A.L.R.); marco.sgarbanti@iss.it (M.S.); 5 Department of Biochemical Sciences, “Sapienza” University of Rome, 00161 Rome, Italy; Luciana.mosca@uniroma1.it; 6 Department of Clinical Medicine, Policlinico Umberto I, “Sapienza” University of Rome, 00161 Rome, Italy; elisa.biliotti@inmi.it; 7 Medical Statistics and Molecular Epidemiology Unit, University Campus Bio-Medico of Rome, 00161 Rome, Italy; m.ciccozzi@unicampus.it; 8 Chronic Infectious Diseases Unit, Policlinico Umberto I, “Sapienza” University of Rome, 00161 Rome, Italy; gloria.taliani@uniroma1.it

**Keywords:** HCV infection, HCV/HIV coinfection, DAA direct-acting antivirals, interferon-stimulated gene (ISG) expression, indoleamine 2-3 dioxygenase (IDO) activity, immune activation

## Abstract

Background: Direct-acting antivirals (DAAs) treatment, although highly efficacious for the treatment of hepatitis C virus (HCV) infection, may not completely reconstitute the HCV-mediated dysregulated immune system, especially in patients co-infected with human immunodeficiency virus (HIV) and HCV. Objectives: We aimed to evaluate the impact of HCV eradication following DAA therapy on the immune system and liver disease improvement through comparative monitoring of 10 HCV mono-infected and 10 HCV/HIV co-infected patients under combined antiretroviral therapy (cART). Early and late longitudinal phenotypic changes in peripheral blood mononuclear cell (PBMC) subsets, T-cell activation, differentiation and exhaustion, as well as inflammatory biomarkers, indoleamine 2-3 dioxygenase (IDO) activity, and liver stiffness, APRI and FIB-4 scores were assessed. Materials and Methods: Samples were obtained at baseline (T0), week 1 (T1), week 2 (T2), week 12 (T3, end of treatment, EOT), and month 9 (T4, end of follow-up, 36 weeks post EOT). Results: All patients achieved a sustained virological response (SVR 12) after DAA treatment. Overall, changes of the T-cell immune phenotypes were greater in HCV/HIV co-infected than in HCV mono-infected, due to an increase in CD4+ and CD8+ T-cell percentages and of CD8+ T-cell activation and memory markers, in particular at the end of follow-up. On the other end, HCV mono-infected showed changes in the activation profile and in the memory CD4+ T-cell compartment. In HCV/HIV co-infected, a decrease in the IDO activity by DAA treatment was observed; conversely, in HCV mono-infected, it resulted unmodified. Regarding inflammatory mediators, viral suppression was associated with a reduction in IP-10 levels, while interferon regulatory factor (IRF)-7, interferon (IFN)-β, and interferon (IFN)-γ levels were downregulated during therapy and increased post therapy. A decrease in liver stiffness, APRI, and FIB-4 scores was also observed. Conclusions: Our study suggests that, although patients achieved HCV eradication, the immune activation state in both HCV mono-infected and HCV/HIV co-infected patients remains elevated for a long time after the end of DAA therapy, despite an improvement of liver-specific outcomes, meanwhile highlighting the distinct immunophenotypic and inflammatory biomarker profile between the groups of patients.

## 1. Introduction

Hepatitis C virus (HCV) infects approximately 3% of the world population and establishes chronic infection in most patients, while 15–25% of patients with acute HCV spontaneously clear the virus [1]. Given shared routes of transmission, HCV infection occurs in 10–30% of individuals infected with human immunodeficiency virus (HIV) under combined antiretroviral therapy (cART), resulting in accelerated chronic hepatitis and development of end-stage liver disease compared to HCV mono-infected subjects [2]. In addition, HIV can be directly involved in liver fibrosis development by activating hepatic stellate cells or promoting pro-inflammatory cytokine secretion [3,4,5]. In turn, HCV infection leads to an increase in the production of inflammatory cytokines and chemokines, giving rise to a permanent activation of the innate immune system and to a state of chronic inflammation with consequent progressive T-cell exhaustion and immune dysregulation [6,7]. Notably, the state of chronic T-cell activation during HCV/HIV coinfection is, in part, driven by a persistent and inadequate regulation of type I and II endogenous interferons (IFN)s that may substantially contribute to the immune dysfunctions related to AIDS progression [8].

Rapid and sustained HCV-RNA clearance by direct-acting antiviral (DAA) therapy is accompanied by changes in hepatic interferon (IFN) and in IFN-stimulated genes (ISGs) expression profiles and by re-modulation and rebalancing of IFN signaling in blood and liver [9,10]. Thus, it is conceivable that HCV clearance by treatments leads to restoring an antiviral state that may interfere with several aspects of HIV-1 replication in HCV/HIV coinfection [11,12,13]. Rapid control of HCV by DAA treatment influences both virologic and immunological control of HIV infection by decreasing immune activation, improving liver-specific outcomes, and restoring specific immune responses against HIV, thus ameliorating the efficacy of antiretroviral therapy [9,11,12,13,14].

During chronic HCV/HIV coinfection, expression of inflammatory biomarkers has been found to correlate with clinical outcomes because of the influence of both viruses on the progression of the disease [13,14,15,16,17,18]. Among these, IDO activity has been considered an inflammation-related marker for HCV and HIV-1 disease progression [19,20,21,22,23]. Defining how inflammation and chronic immune activation interact over the course of treatment is critical; therefore, an evaluation of the effect of DAA regimens on the dynamics of inflammatory biomarkers during HCV and HCV/HIV infections is currently under investigation.

Nowadays, little is known about whether and to what extent immunological alterations induced by HCV are fully reversible upon virus clearance by DAA therapy in HCV mono- as compared to HCV/HIV co-infected patients [15,16,17]. An early report showed that in HCV mono- and HCV/HIV co-infected patients, the frequency of total CD4+ and CD8+ T cells producing IFN-gamma, IL-17, and IL-22 decreased 12 weeks after DAA treatment, whereas no change in T-cell activation was observed at the same time point compared to the baseline [15]. Conversely, a larger study reported a reduction in total activated (HLA-DR+ and CD38+) CD4+ and CD8+ T cells in both groups of patients one-year post SVR [16]. A more recent study showed a significant decrease in plasma biomarkers and gene expression related to antiviral/inflammatory response, particularly in levels of several chemokines and ISGs in HCV/HIV-co-infected patients; however, complete normalization of the immune system was not achieved, as observed by comparison with HCV and HIV mono-infected patients [17]. The studies mentioned above evaluated the effects of treatments on T-cell activation and immune system-related plasma biomarkers at SVR or for short time follow-up with discordant results.

Longer follow-up is also required to answer the important question of whether the inflammatory parameters decline over time in successfully DAA-treated patients and whether long-term restoration of the interferon system is achieved after HCV clearance. Immune recovery could result in liver improvements as measured by the fibrosis stage. Moreover, identifying immune differences between HCV mono- and HCV/HIV co-infected patients may shed light on the immunopathology that results in impaired ability to clear HCV infection and liver disease progression in HCV/HIV co-infected patients, helping to understand the relationship between HCV/HIV coinfection and progression of HIV disease. Finally, suppression of HCV replication by DAA therapy may confer a higher efficacy to cART therapy by reducing immune activation in HCV/HIV coinfection, therefore being associated with improved clinical management of HCV/HIV patients under suppressive ART.

To gain a deeper insight into the link between T-cell responses, inflammation, ISG gene expression, and metabolism in chronic HCV infection and/or HCV/HIV coinfection, we aimed at estimating early and long-term follow-up post SVR changes of several immune parameters to determine the long-term effectiveness of DAA therapy on the restoration of immune homeostasis in infected patients.

HCV eradication following DAA therapy could promote an improvement or normalization in peripheral immune biomarkers related to lymphocyte subpopulations, in inflammation, metabolic biomarkers, and gene expression profile in HCV mono-infected and in HCV/HIV co-infected patients. To this aim, the role of DAA regimens on the restoration of immune dysfunction, the dynamic changes of lymphocyte subsets, and the levels of T-cell phenotypes (including activation, exhaustion, and memory subsets) in successfully treated HCV mono- and HCV/HIV co-infected patients were investigated. The biological correlates associated with antiviral treatment responses, such as type I and II IFNs pathway expression genes and IDO activity, were also explored. Furthermore, clinical benefits of HCV clearance on disease progression were evaluated by a long-term, post-SVR follow-up in terms of improvement of liver fibrosis data, monitoring reduction in liver stiffness, and AST to platelet ratio index (APRI) and fibrosis-4 (FIB-4) scores.

## 2. Materials and Methods

### 2.1. Ethics

The study protocol was approved by the ethics committee of the Policlinico Umberto I, Rome, Italy (protocol no. 4795, 2/13/2018). Data were processed using unique identifiers to ensure confidentiality. Analysis and treatment of personal data were conducted according to the Italian law 196/2003 and the EU regulation of the European Parliament and the European Council no. 2016/679. All study participants provided their informed consent to participate, and all procedures were followed in accordance with the Declaration of Helsinki (authorization no. 9/2014—General Authorization to Process Personal Data for Scientific Research Purposes of the Italian Data Protection Authority).

### 2.2. Study Design and Sample Collection

This prospective study was carried out at the Policlinico Umberto I, University of Rome “Sapienza”. Patients were distributed in two groups: HCV mono-infected (10) and HCV/HIV co-infected (10).

The inclusion criteria were: male or female, at least 18 years of age, plasma HCV-RNA positivity, confirmed chronic hepatitis C (CHC), no previous anti-HCV therapy, for HCV/HIV co-infected patients stable combined antiretroviral therapy (cART) ≥ 12 months with plasma HIV-1 RNA copies/mL <37 for at least 6 months prior to starting DAA therapy and CD4 T-cell count > 500 cells/μL. The exclusion criteria were: cirrhosis, HBsAg positivity, malignancy, opportunistic infections, autoimmune diseases, prior or present alcohol abuse, pregnancy or breastfeeding, and any chronic liver disease unrelated to HCV.

Based on EASL Clinical Practice Guidelines and access to HCV treatment based on the indication of the Italian Medicines Agency, the patients then underwent two different DAA regimens for 12 weeks. Treatment efficacy was measured as a sustained virologic response (SVR12), defined as HCV RNA < 15 IU/mL. Blood samples collection was scheduled using the following timing: T0 = baseline (the day when the DAA treatment starts); T1 = after 1 week of therapy; T2 = after 2 weeks of therapy; T3 = end of therapy (EOT: 12 weeks of therapy); T4 = end of follow-up (36 weeks after EOT).

Healthy participants (10) were included as controls (HC) for evaluating differences of HCV mono-infected and HCV/HIV co-infected patients with respect to the normality in peripheral blood biomarkers. HC were age (52 (50–65)) and sex-matched (50% male to female ratio), HIV and HCV-seronegative, and they met all the exclusion criteria.

### 2.3. Viral Loads, Clinical Parameters, and Fibroscan Assessment

Plasma HIV-1 and HCV RNA levels were measured using Versant kPCR (Siemens Healthcare Diagnostic Inc., Tarrytown, NY, USA). The limit of detection was 37 copies/mL for HIV-1 and 15 IU/mL for HCV. Liver stiffness (kPa) and values of aspartate aminotransferase (AST), alanine aminotransferase (ALT), and gamma-glutamyl transferase (GGT) were assessed using transient elastography by Fibroscan and blood test, respectively. To determine the degree of hepatic fibrosis, we also used the FIB-4 score and APRI. According to the Child–Pugh classification system, all patients were Child–Pugh-A. All patients were monitored for liver fibrosis by transient elastography, FIB-4, and APRI at T0 and T4.

### 2.4. Immunophenotypic Analysis

Whole blood samples were collected in EDTA tubes by peripheral venipuncture. Peripheral blood mononuclear cells (PBMCs) were isolated using Ficoll-Paque (GE Health Care Life Sciences, Uppsala, Sweden), washed twice in PBS, and stained with commercial fluorochrome-conjugated anti-human monoclonal antibodies (mAbs): anti-CD4, anti-CD8, anti-CD20, anti-CD16, anti-CD25, anti-CD28, anti-CD38, anti-CD45RA, anti-CD45R0, anti-CD56, anti-CD69, anti-HLA-DR, and anti-programmed death receptor-1 (PD-1) (Becton-Dickinson, Holdrege, NE, USA) as previously described [24]. After incubation with the antibodies for 30 min at 4 °C, cells were washed and fixed in 1% paraformaldehyde (Sigma Chemical Co., St. Louis, MO, USA). Samples were acquired by FACSCanto flow cytometer and analyzed with the BD FACSDiva Software (BD Biosciences, San Jose, CA, USA).

### 2.5. Quantitative Real-Time Reverse Transcription-PCR and IP-10 Detection

Total RNA isolated from PBMC was extracted using the RNeasy total RNA extraction kit (Qiagen, Waltham, MA, USA) treated with RNase-free DNase (Qiagen) and then reverse transcribed with High Capacity cDNA Reverse Transcription Kit (Applied Biosystems, Waltham, MA, USA), according to the manufacturer’s instructions. cDNA was subjected to quantitative real-time PCR on ABI 7000 sequence detection system (PE Applied Biosystems, Warrington, UK) by using SYBR green PCR master mix (Applied Biosystems). Primers used for quantitative real-time reverse transcription-PCR (qRT-PCR) were MX1: forward (5′-GCCAGGACCAGGTATACAG-3′) reverse (5′-GCTCCTTCAGGAGCCAGA-3′); IFN-γ: forward (5′-TGTAGCGGATAATGGAACTCTTTT) reverse (5′-AATTTGGCT CTGCATTATT-3′), IFN-β: forward (5′-GAGCTACAACTTGCTTGGATTCC-3′) reverse (5′-CAAGCCTCCCATTCAATTCC-3′) IRF-7: forward (5′-AGAGGGCGTTTTATCTTG CG) reverse, (5′-TGGAGCCCAGCATTTTCT CT-3′). Transcript levels were normalized to glyceraldehyde-3-phosphate dehydrogenase (GAPDH) forward (5′-GGGTGTGAACCATGAGAAG-3′), reverse (5′-GCTAAGCAGTTGGTGGTGC-3′) as an endogenous reference gene and expressed as fold increase according to the ΔΔ*C_T_* methods (means ± standard deviations). Relative quantification of gene expression was calculated using the ΔΔCt (Ct, threshold cycle of real-time PCR) method according to the formula: ΔCT = Ct reference − Ct target, ΔΔCt = ΔCt control − ΔCt target, Ratio = 2 − ΔΔCt [25].

Commercially available ELISA kits (Boster Biological Technology, Pleasanton, CA, USA) were used for the quantitative detection of plasma levels of interferon gamma-induced protein 10 (IP-10) according to the manufacturer’s instructions. All samples were tested in duplicate. Data from 5 healthy volunteers were used for comparison.

### 2.6. Measurement of Kynurenine: Tryptophan Ratios

Plasma samples were stored at −80 °C until analysis. An aliquot of 200 μL plasma was treated with perchloric acid at a final concentration of 5%, vortex-mixed for 1 min, stored on ice for 30 min, and then centrifuged at 20,000× *g* for 30 min at 4 °C. A total of 200 μL of clear supernatant were directly injected onto the HPLC system for analysis. The chromatographic apparatus was a Waters HPLC equipped with a 600 pump and pump controller, an XBridge C18 column (reverse phase, 4.6 mm × 150 mm, 3.5 µm particle size, with a 10 mm guard column of the same material) thermostated at 25 °C, an autosampler mod.717, and a UV-Vis photodiode array detector mod. 2996. Chromatographic elution was performed in isocratic conditions with a mobile phase consisting of 15 mM sodium acetate buffer (pH 4.0) and acetonitrile (95:5, *v*/*v*) at a flow rate of 1 mL/min. The eluate was monitored at 360 nm for Kyn and at 280 nm for Trp. Kyn and Trp amount in plasma samples were calculated on the basis of a calibration curve obtained by using appropriate standards. The stock solutions of Kyn and Trp were dissolved in methanol at the concentrations of 5 and 20 mM, respectively, and stored at −20 °C. A series of mixed standard working solutions with different concentrations were obtained by further dilution of each standard stock solution with the mobile phase immediately before use. IDO1 activity was calculated as the [Kyn](µmol/L)/[Trp](µmol/L) ratio.

### 2.7. Statistical Analysis

Quantitative variables were summarized using median and range. Frequency distributions were presented for categorical variables. Comparisons of quantitative variables between and among different groups were performed by using nonparametric tests (Mann–Whitney test and Kruskal–Wallis test, respectively). Changes at T4 from T0 for each group were analyzed using the Wilcoxon signed rank-sum test for paired data. Fisher’s exact and 2-tailed t-tests were carried out to test differences in HCV mono-infected and HCV/HIV co-infected patients with regard to qualitative variables. Multilevel linear regressions with an interaction term between the type of infection and time points and a random effect at the patient level were carried out to investigate changes in peripheral immune phenotype, systemic inflammation, and type-I interferons signaling over time in the HCV mono- and HCV/HIV co-infected patients. Z-scores of IRF-7, IFN-β, IFN-γ, and HCV RNA were calculated. A linear structural equation model by type of infection and patient clustered standard errors with IRF-7, IFN-β, IFN-γ, and HCV RNA as dependent variables, and time points as the independent variable was carried out to investigate the equality of coefficients between IRF-7, IFN-β, IFN-γ z-scores, and HCV RNA z-score. In this model, the dependent variables were allowed to be correlated. The association between IP10 and HCV RNA was investigated by a piecewise linear structural equation model with individual-level random effects. Statistical analyses were carried out two-sided with a 0.05 significance level, using SAS^®^ (Version 9.4, SAS Institute Inc., Cary, NC, USA) and STATA (Version 16.1, Stata Corp LLC, College Station, TX, USA).

## 3. Results

### 3.1. Study Population Characteristics

**Figure 1:**
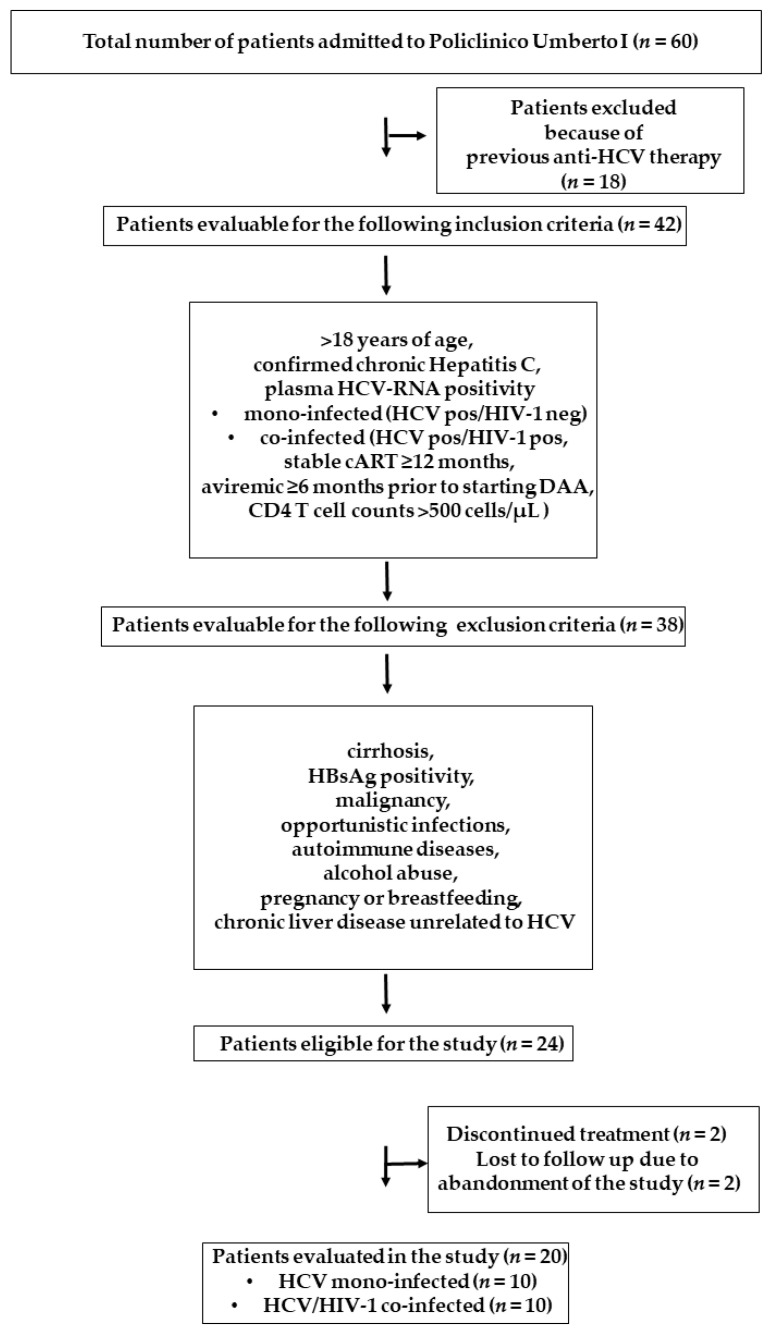
Flow diagram of patients recruited in the study.

A flow diagram describing the selection of the patients included in this study is shown in Figure 1.

The main characteristics of patients at the time of inclusion in the study are shown in Table 1. The median age was comparable among the groups HCV mono- and HCV/HIV co-infected (median age(range): 52.5 (48–66), 50.5 (48–60)). The number of males in HCV/HIV co-infected was not significantly higher as compared to the HCV mono-infected patients (*p* = 0.141), and before DAA therapy, the HCV-RNA plasma level in HCV mono-infected was not significantly lower than that in the HCV/HIV co-infected group (*p* = 0.0806). Regarding the HCV genotype 1, no statistical difference was observed between HCV mono- and HCV/HIV co-infected subjects (*p* = 0.475). The median duration of cART in HCV/HIV co-infected patients was 15 years prior to starting DAA therapy. All patients were successfully treated with anti-HCV DAA combinations achieving SVR12 and had undetectable serum HCV RNA at the end of therapy. All patients were followed for 36 weeks after EOT. At baseline (T0), liver transaminases ALT, AST, and GGT values were lower in HCV mono-infected patients compared to HCV/HIV co-infected patients, although the differences did not reach statistical significance (ALT: 65.6, 39–154 and 83, 25–383); (AST: 48, 34–117 and 68.5, 16–221); (GGT: 67, 16–442 and 75, 31–209), respectively (Table 1). Liver stiffness, APRI and FIB-4 were similar among HCV mono-infected and HCV/HIV co-infected patients (stiffness: 10.1, 4.6–12.5 and 10, 4–14), (APRI: 0.7, 0.5–1.1 and 0.7, 0.5–1.4), (FIB-4: 1.8, 1.7–2.4) respectively (Table 1). After DAA therapy, ALT and AST levels significantly decreased both in HCV mono- (ALT: 18.5, 13–23, *p* = 0.0020; AST: 16, 9–22, *p* = 0.0020) and HCV/HIV-1 co-infected (ALT: 20, 8–45, *p* = 0.0059; AST: 20, 15–65, *p* = 0.0195), whereas GTT level showed significant decline only in HCV patients (GTT: 19, 11–48, *p* = 0.0039) (Table 1). A reduction in liver stiffness (HCV mono-infected *p* = 0.0050; HCV/HIV-1 co-infected *p* = 0.0130) and an improvement in fibrosis scores were observed in both groups; however, at T4, only HCV mono-infected significantly decreased both APRI and FIB-4 scores (*p* = 0.0020 for both) (Table 1).

### 3.2. Longitudinal Changes in Peripheral Immune Phenotype after DAA Treatment

**Figure 2:**
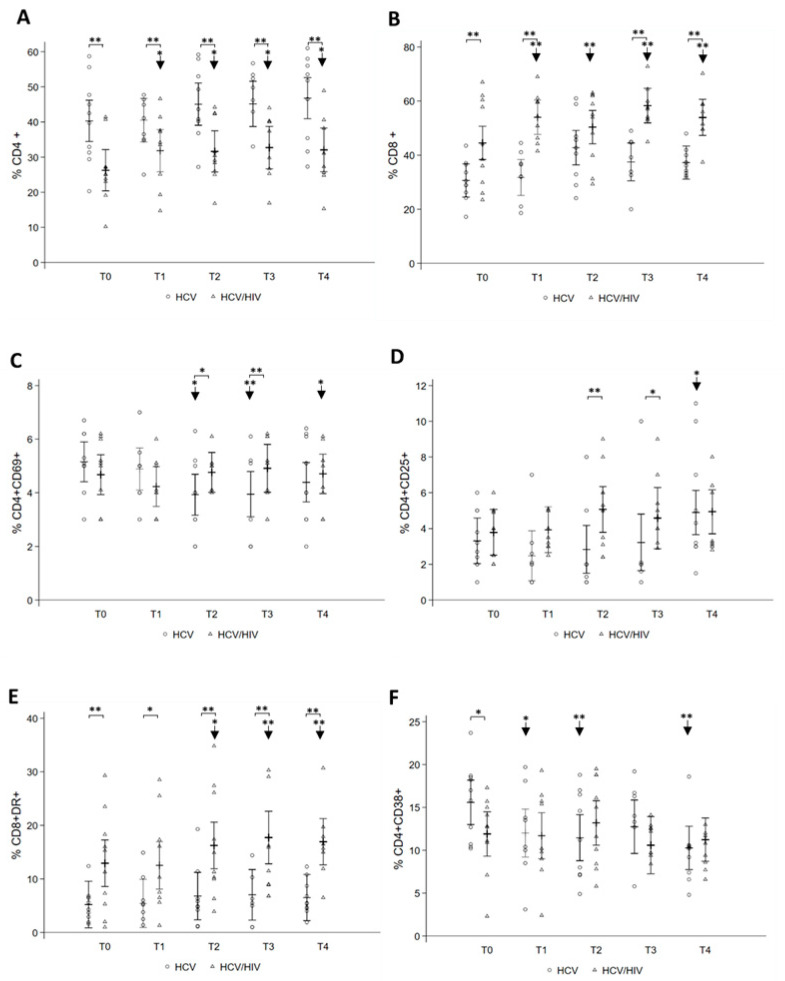
Longitudinal analysis of CD4+ and CD8+ T cells and of CD4+/CD8+ T-cell subsets before and after DAA therapy. Whole blood T-cell subsets of HCV mono-infected and HCV/HIV co-infected were compared from T0 up to T4. Levels of CD4 (**A**), CD8 (**B**), and different CD4 and CD8 activation subsets on the basis of CD69 (**C**), CD25 (**D**), HLA-DR (**E**), and CD38 (**F**) expression are shown. Statistically significant differences over time are marked by an arrow and asterisk, and differences between the two groups of patients by an asterisk. One asterisk corresponds to *p* < 0.05, two asterisks to *p* < 0.001. Data are shown as hollow circles (HCV) and triangles (HCV/HIV). Multilevel linear regressions predicted estimates are reported as mean and 95% CI.

To investigate the changes in CD4+ and CD8+ lymphocyte immune phenotypes following successful DAA treatment in HCV mono- and HCV/HIV co-infected patients, we measured expression of activation markers CD69, CD25, HLA-DR, CD38 and CD28, exhaustion marker PD1, and naïve/memory markers CD45RA/CD45RO from baseline and at each time point over time. At the baseline, CD4+ T-cell number was significantly lower in HCV/HIV co-infected as compared to the other group and increased significantly during the following visits (Figure 2A) from 26.25% (95% CI: 20.37; 32.13) at T0 to 32.71% (95% CI: 26.91; 38.51) at T4, whereas in HCV mono-infected it became statistically significantly higher at the end of follow-up changing from 40.31% (95% CI: 34.43; 46.19) at T0 to 46.51% (95% CI: 40.72; 52.30) at T4 (Figure 2A). The percentage of CD8+ T cells, that was markedly higher in HCV/HIV co-infected throughout the period of observation (Figure 2B), significantly increased from 44.51% at T0 (95% CI: 38.32; 50.69) to 54.54% at T4 (95% CI: 48.51; 60.57) in HCV/HIV co-infected, and from 30.66% at T0 (95% CI: 24.47; 36.84) to 42.74% at T2 (95% CI: 36.32; 49.17) and 37.60% at T4 (95% CI: 31.56; 43.64) in HCV mono-infected (Figure 2B).

Regarding immune activation markers, at the baseline, in HCV mono-infected, CD4+CD69+ T-cell levels significantly declined at T2 (3.93%; 95% CI: 3.16; 4.69) and T3 (3.94%; 95% CI: 3.10; 4.79), showing significantly lower levels than HCV/HIV co-infected, then they increased again after therapy (4.39%; 95% CI: 3.65; 5.12) without, however, returning to baseline values (5.15%; 95% CI: 4.40; 5.89) (Figure 2C), whereas no difference was observed in the CD8+CD69+ T-cell levels. CD4+CD25+ were higher in HCV/HIV co-infected as compared to HCV mono-infected; however, in the latter group, a statistically significant increase in this cell subset was present at T4 (Figure 2D).

Analysis of HLA-DR marker showed that CD8+HLA-DR+ T-cell levels significantly increased over time in HCV/HIV co-infected from 12.93% (95% CI: 8.58; 17.28) to 16.94% (95% CI: 12.62; 21.26) after treatment, whereas no changes were observed in HCV mono-infected, who exhibited lower levels than HCV/HIV co-infected (Figure 2E). CD4+HLA-DR+ level was similar in both groups throughout the study.

At the baseline, higher levels of CD4+CD38+ cells in HCV mono- as compared to HCV/HIV co-infected were observed, that significantly decreased over the course of treatment from 15.59% (95% CI: 13.00; 18.18) at T0 to 10.27% (95% CI: 7.73; 12.80) at T4 (Figure 2F), whereas HCV/HIV co-infected showed higher values of both CD4+CD38+ and CD8+CD38+ during the study (Figure 2F).

**Figure 3:**
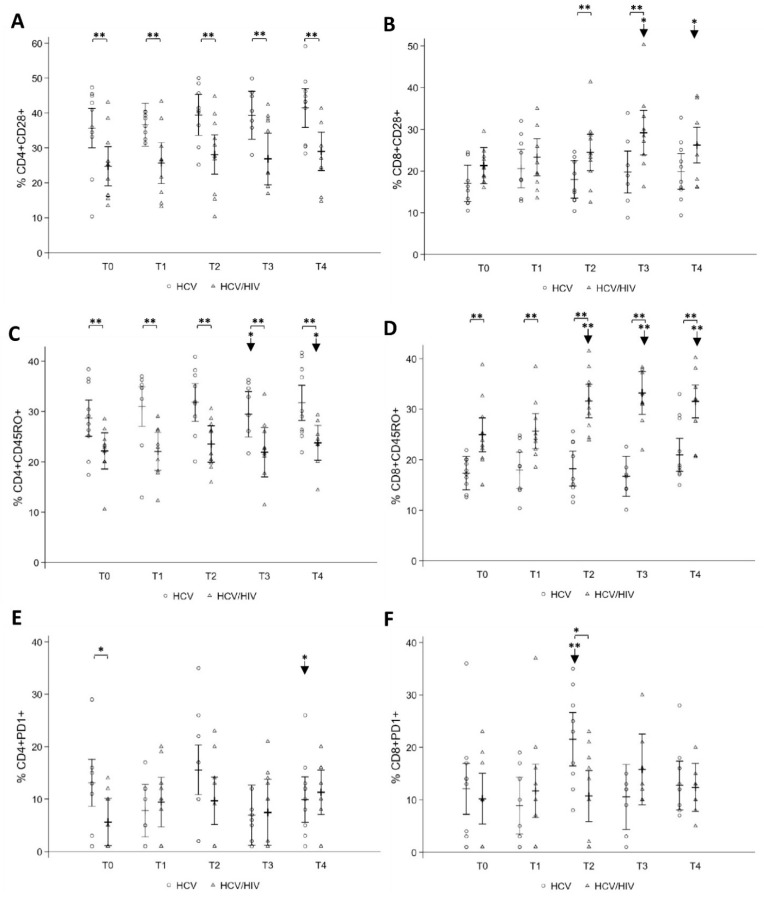
Longitudinal analysis of CD4+/CD8+ T-cell subsets before and after DAA therapy. Whole blood T-cell subsets of HCV mono-infected and HCV/HIV co-infected were compared from T0 up to T4. Levels of expression of CD28 (**A,B**), CD45RO (**C**,**D**), and exhaustion PD-1 (**E**,**F**) markers are shown. Statistically significant differences over time are marked by an arrow and asterisk, and differences between the two groups of patients by an asterisk. One asterisk corresponds to *p* < 0.05, two asterisks to *p* < 0.001. Data are shown as hollow circles (HCV) and triangles (HCV/HIV). Multilevel linear regressions predicted estimates are reported as mean and 95% CI.

Analysis of CD28, a molecule essential for both immune cell activation and proliferation of naïve and memory T cells, was also assessed. No variations in CD4+CD28+ expression were observed in both groups by treatment outcome (Figure 3A); however, HCV mono-infected showed significantly higher levels than HCV/HIV co-infected throughout the period of the study (Figure 3A). Regarding CD8+CD28+ expression, after treatment, a statistically significant increase at T3 (29.22%; 95% CI: 23.89; 34.56) and T4 (26.23%; 95% CI: 21.95; 30.50) from T0 (21.30%; 95% CI: 16.96; 25.64) was observed in HCV/HIV co-infected, who also showed higher levels as compared to HCV mono-infected (Figure 3B).

The effect of DAA treatment on naïve and memory (CD45RA+/CD45RO+) T-cell subsets was also determined. In HCV mono-infected, CD4+CD45RO+ were higher than in HCV/HIV co-infected throughout the period of the study and significantly increased from the baseline (28.66%; 95% CI: 25.07; 32.25) to T2 (31.80%; 95% CI: 28.03; 35.56) and T4 (31.72%; 95% CI: 28.24; 35.20) (Figure 3C). On the contrary, CD8+CD45RO+ significantly increased in HCV/HIV co-infected from T0 (24.91%; 95% CI: 21.57; 28.25) to end of the study (31.52%; 95% CI: 28.25; 34.80) (Figure 3D). Expression of CD4+CD45RA+ and CD8+CD45RA+ did not differ by treatment outcome in both groups of patients.

When the immune exhaustion PD-1 marker was assessed, at T0, HCV mono-infected displayed significantly higher CD4+PD-1+ T-cell levels compared to HCV/HIV co-infected, and at T4, a significant reduction was observed only in the first group (Figure 3E). In contrast, CD8+PD-1+ T-cell levels increased at T2, and at the same time point, were higher than those expressed in HCV/HIV co-infected (Figure 3F).

No significant differences were observed in CD20+ and CD16+CD56+ cell subsets over the course of the study, either in HCV or HCV/HIV patients.

### 3.3. Changes in Systemic Inflammation after DAA Treatment

**Figure 4:**
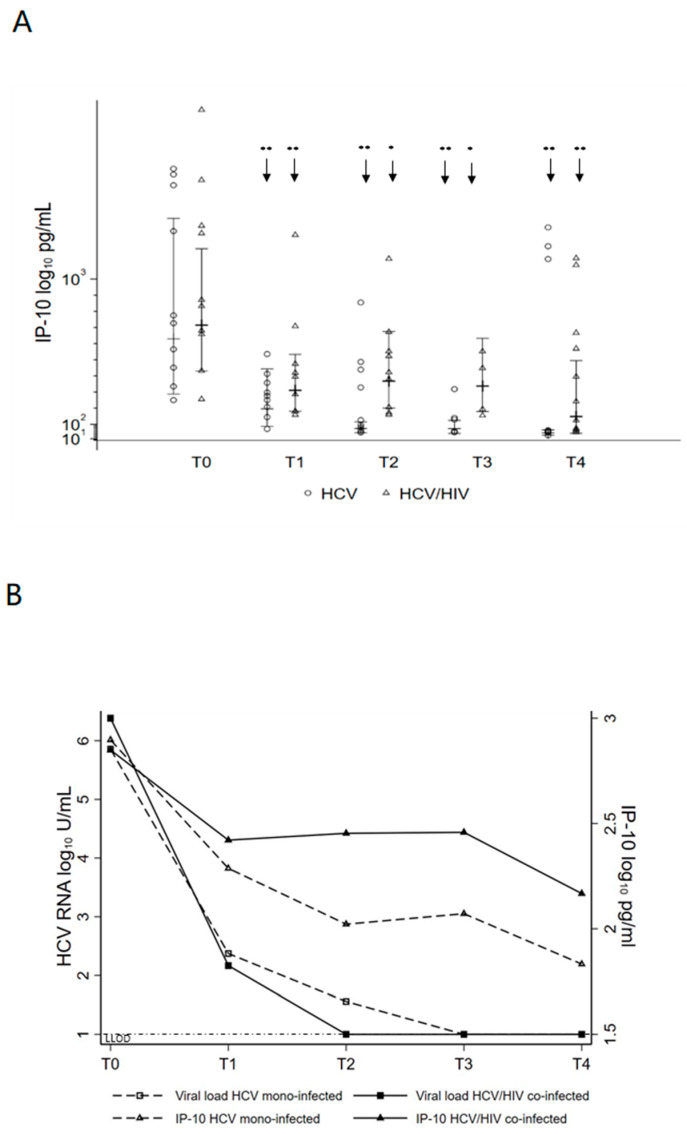
Serum IP-10 levels normalize with DAA treatment and correlate with viral kinetic decline. (**A**) Serum IP-10 protein levels upon treatment. Statistical analysis showed a significant change over time regardless of treatment outcome that differed between HCV mono-infected and HCV/HIV co-infected. This difference disappeared at the end of follow-up. (**B**) Serum IP-10 levels at baseline and during treatment mirrored changes in viral load. LLOD, lower limit of detection for viral load. (**A**,**B**) Stepwise linear structural equation model predicted estimates are shown for the HCV mono-infected and the HCV/HIV co-infected. Statistically significant differences over time are marked by an arrow and asterisk. One asterisk corresponds to *p* < 0.05, two asterisks *p* < 0.001.

To investigate biologic correlates of treatment response in mono-infected and co-infected patients, plasma levels of the IFN-inducible cytokine IP-10, considered a biomarker for clearance of HCV [26], expression of IFN-stimulated genes (ISGs) IRF7 and MX1, and of IFN-β and IFN-γ were evaluated. At baseline, IP-10 plasma levels were similar in HCV mono-infected and HCV/HIV co-infected (higher than HC median 81.3, ranges 40.7–261.3) and significantly decreased by treatment outcome during therapy, from T0 (652.07; 95% CI: 361.30; 1176.85) to T4 (102.85; 95% CI: 25.96; 407.46) in HCV mono-infected and from T0 (1216.39; 95% CI: 900.26; 1643.53) to T4 (168.11; 95% CI: 64.26; 439.77) in HCV/HIV co-infected (Figure 4A). Moreover, viral kinetic and IP-10 decline were significantly correlated in both HCV mono-infected and HCV/HIV co-infected (Figure 4B).

**Figure 5:**
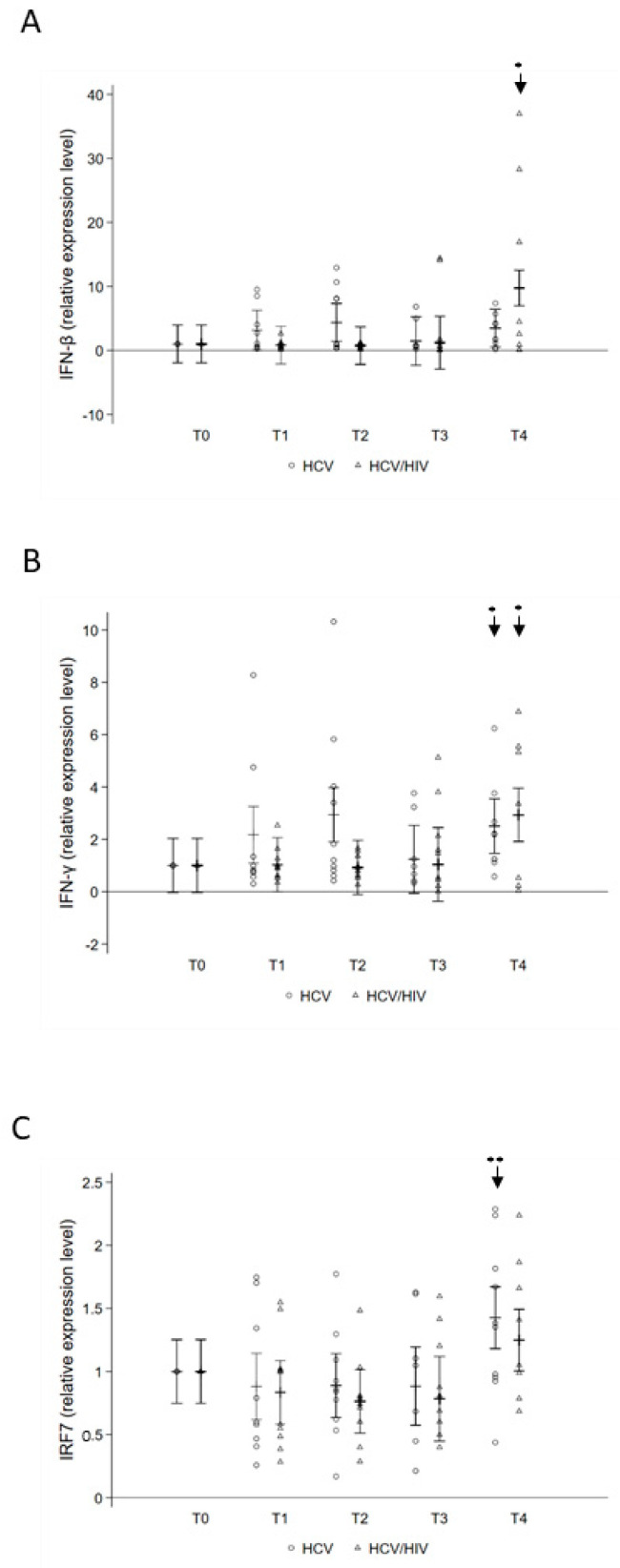
Treatment-induced changes in expression of IFNs and of IRF7-IFN gene. Total RNA was purified, at different time points, from PBMC of 10 HCV mono-infected and 10 HCV/HIV co-infected and analyzed for gene expression of (**A**) IFNβ, (**B**) IFNγ, and of (**C**) IRF7 by quantitative reverse transcription-PCR by normalizing with GAPDH mRNA. Levels from uninfected cells were set as the basis of comparative results. Multilevel linear regressions predicted estimates are reported as mean and 95%CI. Statistically significant differences over time are marked by an asterisk. One asterisk corresponds to *p* < 0.05, two asterisks *p* < 0.001.

At the baseline, no differences in IRF7, IFN-β, and IFN-γ expression levels were found between HCV and HCV/HIV infected individuals (Figure 5).

Over the course of treatment, both IFN-β and IFN-γ gene expression significantly increased at T4 in HCV/HIV co-infected and IFN-γ in HCV mono-infected (Figure 5A,B). Conversely, IRF7 gene expression showed a significant increase at T4 only in HCV mono-infected patients (Figure 5C). No statistical significance variations were observed in MX1 gene expression levels.

### 3.4. Kynurenine-to-Tryptophan Ratio in HCV Mono- and HCV/HIV Co-Infected Patients

**Figure 6:**
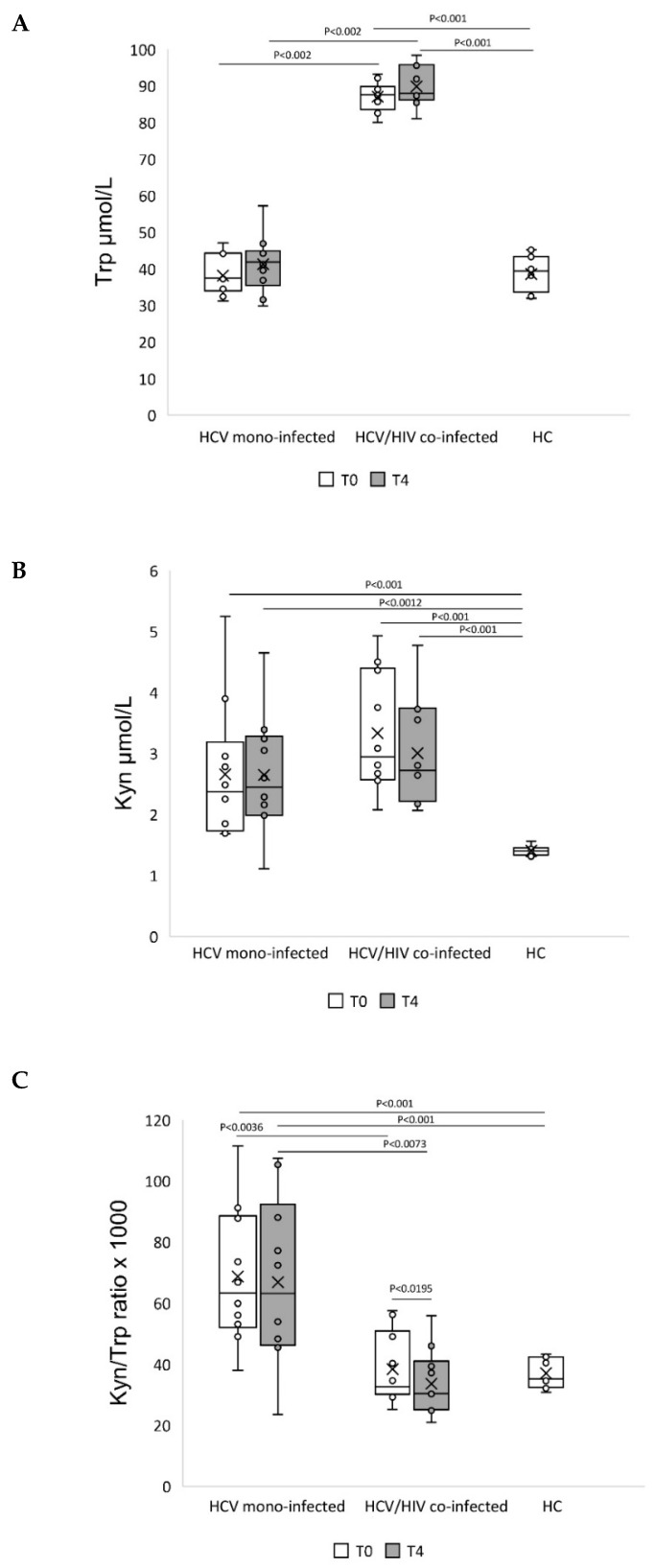
Different tryptophan catabolism outcome in DAA-treated HCV mono-infected and HCV/HIV co-infected. Plasma levels of (**A**) Kyn (µmol/L), (**B**) Trp (µmol/L), and (**C**) IDO enzyme activity defined as the Kyn/Trp ratio in HCV mono-infected, HCV/HIV co-infected, and healthy control (HC). Data are presented using a box plot. Comparison between groups was performed using the Wilcoxon–Mann–Whitney test, comparison among groups was performed using the Kruskal–Wallis test while change at T4 from T0 in each group was analyzed using the Wilcoxon signed rank-sum test.

We assessed Trp catabolism in HCV mono- and HCV/HIV co-infected patients at baseline (T0) and at the end of follow-up (T4). The concentrations of Trp, Kyn, and the Kyn/Trp ratio were compared among healthy controls, mono-infected, and co-infected patients. Before DAA treatment, HCV/HIV co-infected (C) displayed significantly higher Trp levels compared to HCV mono-infected (M) and HC, which instead showed similar values (C: 87.51 (83.83–89.06)) vs. M: 37.44 (34.48–44.16) and HC: 39.34 (33.58–43.33) μmol/L; Figure 6A).

Although higher Kyn levels were seen in the HCV/HIV co-infected than in the HCV mono-infected, the difference was not statistically significant (C: 2.95 (2.57–4.36) vs. M: 2.37 (1.75–2.95) μmol/L. Conversely, levels of Kyn were significantly higher in HCV/HIV co-infected and HCV mono-infected compared to HC (HC: 1.42 (1.33–1.45) μmol/L; Figure 6B). Correspondingly, also IDO activity (Kyn/Trp) was also significantly elevated in HCV mono-infected subjects compared to other groups (M: 63.27 (53.11–87.70) vs. C: 32.66 (30.47–49.09)), HC: 35.15 (32.38–42.43) μmol/L, Figure 6C). At T4, HCV mono-infected maintained levels of Trp (T0 M: 37.44 (34.48–44.16) vs. T4 M: 41.79 (36.82–44.16) μmol/L) and Kyn (T0 M: 2.37 (1.75–2.95) vs. T4 M: 2.44 (1.99–3.24) μmol/L) similar to pre-treatment, while HCV/HIV co-infected showed similar levels of Trp (T0 C: 87.51 (83.83–89.06) vs. T4 C: 87.94 (86.42–95.54) μmol/L) and not statistically significant lower levels of Kyn (T0 C: 2.95 (2.57–4.36) vs. T4 C: 2.72 (2.23–3.73) μmol/L). After treatment, no change of IDO activity was detected in HCV mono-infected (T0 M: 63.27 (53.11–87.70) vs. T4 M: 63.12 (46.53–88.00), HC: 35.15 (32.38–42.43) μmol/L) (Figure 6C). This group, despite Trp levels comparable to HC both at T0 and at T4, showed higher levels of Kyn compared to HC and consequently of IDO activity, indicating that DAA therapy and clearance of HCV did not normalize the Kyn pathway of Trp catabolism. Conversely at T0, HCV/HIV co-infected displayed lower IDO activity as compared to HCV mono-infected (T0 C: 32.66 (30.47–49.09) vs. M: 63.27 (53.11–87.70) μmol/L), and after DAA therapy a significantly decrease at T4 (T0 C: 32.66 (30.47–49.09) vs. T4 C: 30.37 (25.23–39.33), HC: 35.15 (32.38–42.43) μmol/L) (Figure 6C). Thus, in HCV/HIV co-infected, Trp catabolism was characterized by significantly higher plasma Trp and Kyn levels despite other groups and lower IDO activity after treatment.

## 4. Discussion

Over past years, it has been established that chronic antigenic stimulation during persistent infections with HCV or HIV is associated with continuous activation and impaired function of the immune system [8]. However, it is not fully understood whether, following DAA therapy, HCV eradication leads to a restoration of both innate and adaptive immune responses and of homeostasis of lymphocyte population, previously dysregulated by the virus [27,28,29,30,31], and to a reduction in liver inflammation that might result in improvement of liver fibrosis and HCV disease-related prognosis [32,33,34]. The significance of the estimated reduction in fibrosis measured non-invasively is of pivotal clinical importance. In some studies, transient elastography was assumed to predict fibrosis regression, while in others, it has been found that transient elastography improvements could be overstated when compared with the histologic staging [32].

Our results, although limited due to the small sample size, show that after DAA therapy, all patients experienced a significant reduction in liver stiffness from onset to long-term, post-SVR follow-up. However, a statistically significant improvement in the APRI and FIB-4 scores was seen only in HCV mono- as compared to HCV/HIV co-infected patients. In HCV mono-infected, the observed long-term fibrosis’ score decrease after DAA treatment, may display fibrosis regression as also quantified by APRI, FIB-4, and liver stiffness values. Conversely, in HCV/HIV, co-infected fibrosis regression was predicted only by an improvement in liver stiffness, while APRI and FIB-4 measures did not achieve statistical significance. This different pattern at long-term post SVR could likely reflect an immune recovery in HCV mono-infected, due to reduced migration of lymphocytes to the inflamed liver resulting in regression of fibrosis, and differently in HCV/HIV co-infected, a slight decrease in liver inflammatory activity and, as a consequence, a minor improvement of fibrosis scores APRI and FIB-4 after treatment. Of note, no association was found between liver stiffness, APRI, FIB-4, and peripheral blood biomarkers.

Changes of T-cell immune phenotypes were much more remarkable in HCV/HIV co-infected than in HCV mono-infected [35]. An improvement of the CD4+ compartment was observed just after one week of treatment and persisted for a long time after HCV cure in HCV/HIV co-infected. This result is of great importance and points out that DAA treatment may represent a high priority for this population.

In fact, both HIV and HCV viruses can affect CD4+ cell count, particularly in HCV/HIV co-infected patients, and DAA treatment may be an opportunity to restore the CD4+ T-cell compartment. After DAA therapy initiation, the rapid increase in peripheral CD4+ T-cell number combined with a decline of HCV viral load and of hepatic transaminase concentrations may suggest an egress of hepatic tissue-resident lymphocytes, following virus clearance and reduction in liver inflammation, as HCV-specific T cells are no longer required in the liver parenchyma to suppress viral replication [35,36]. Thus, inhibition of HCV replication by DAA therapy could result in the reappearance of HCV-specific CD4+ T cells in the peripheral blood after elimination of the persistent antigen [35,36].

Reasons for the progressive increase in CD8+ observed during treatment are unclear, although it is possible that HCV clearance itself leads to increased CD8+ T lymphocyte levels, as it is described that persistent infection with HCV results in CD8 T-cell exhaustion, with impaired proliferation and increased apoptosis [37]. Otherwise, it is also conceivable that after HCV clearance, changes in HIV cytokine balance or cytokine network may occur, triggering the production of pro-inflammatory and/or homeostatic cytokines that, in turn, would stimulate and/or maintain CD8+ T-cell proliferation/activation.

On the other hand, also the fluctuation of the CD8+ T cells observed during the study, and the increase in their levels seen at the end of the follow-up in HCV mono-infected could be explained as a recovery of this lymphocyte subset induced by the virus clearance after treatment.

DAA therapy results in restoring, in HCV mono-infected, T-cell immune phenotype as shown by the significant decline in immune activation and improvements in T-cell subsets with an increase in memory phenotypes. Results evidenced low levels of CD4/CD28, a decrease in CD4/CD69 and CD4/CD38 expression with resolution of T-cells activation, and an increase in CD4/CD45RO largely sustained up to the end of follow-up. The observed decrease in activated CD4+ T-cell subsets from pre-treatment to post SVR suggests that HCV clearance normalizes activated CD4+T-cell levels. Further, DAA treatment, by preventing progressive T-cell exhaustion characteristic of chronic HCV infection, may lead to enhanced memory CD4+ T cells and improved protection against progression to chronicity. In fact, evidence that CD4+ T-cell responses to HCV and the adequate CD4+ T-cell help to CD8+ T-cell subsets play an important role in the outcome of HCV infection has been shown in several studies [30,35,36,37], as well as the fact that perturbing homeostasis of memory T cells may have an important role in disease pathogenesis [35].

A different picture appeared in the CD4+ T-cell compartment of HCV/HIV co-infected during the treatment. Our results demonstrated an overall progressive improvement in CD4+ T-cell percentage; however, no significant influence of DAA therapy in the subset profiles CD28, CD38, or CD45RO was observed. On the other hand, analysis of CD8+ T cells showed important changes such as an increased CD28, CD45RO, and HLA-DR expression without a concomitant decrease in CD38 for long-term follow-up outcome. These data are in contrast with those previously reported by other authors showing a reduction in immune activation induced by treatment [15,38]. The difference could be due to the sample size or the characteristic of the study population (e.g., fibrosis stage, cirrhosis, different anti-HCV treatment, DAA duration for SVR). Najafi Fard et al. reported that there was no significant change from pre-DAA therapy to SVR for activated CD4+ and CD8+ T cells in both HCV mono-infected (*n* = 18) and HCV/HIV co-infected (*n* = 17) patients [15]. Shrivastava et al., in accordance with HCV/HIV co-infected patients of the ERADICATE study (*n* = 50), did not observe any significant decline in the CD38 expression [37].

Regarding T cell exhaustion, our analysis is limited to one marker only of exhausted phenotype and, no significant impact of DAA therapy on PD-1 expression on circulating T cells was detected, as also reported in ERADICATE study [37].

Considering the overall results, it can be stated that, especially in the HCV/HIV co-infected, immune activation may persist for a long time after the end of treatment.

Since HCV infection is associated with a profound activation of the interferon system by induction of interferon-stimulated gene expression, we also asked whether, following DAA, therapy HCV clearance could normalize inflammatory cytokine and chemokine levels. We observed a reduction in expression levels of inflammatory mediators IRF7, IFN-β, and IFN-γ that rebounded to pre-treatment levels in both groups of patients long after DAA therapy. This suggests that DAA-induced HCV clearance does not completely restore the altered inflammatory mediator milieu, at least in those patients who have had chronic HCV infection, as also shown by other groups [30].

Differently from our study, single studies in either the HCV mono-infected or the HCV/HIV co-infected patients have been conducted, including individuals with liver cirrhosis, compensated or advanced, and evaluated at different follow-up time points. Brochado-Kith et al. showed that following DAA therapy in HCV/HIV co-infected patients (*n* = 33) at 36 weeks after SVR, patients with advanced cirrhosis had an improvement in liver disease markers and a significant decrease in plasma biomarkers and gene expression related to antiviral/inflammatory response, particularly in levels of several chemokines and ISGs. However, normalization of the biomarker values was not achieved, as observed by comparison with HCV (*n* = 9) and HIV (*n* = 26) mono-infected patients [17].

Considering IP-10, the higher levels in HCV/HIV co-infected compared to HCV mono-infected patients could be related to HCV infection rather than to HIV replication, which is controlled by cART. It should be noted that DAA therapy has been extremely effective in inhibiting HCV replication, with a consequent reduction in IP-10 levels, in both HCV mono-infected and HCV/HIV co-infected, thus confirming it has an effective prognostic biomarker for the resolution of infection [26].

Trp catabolism has been shown to play a role in both HIV and HCV mono-infection and in HCV/HIV coinfection [19,20,21,22]. In particular, high indoleamine-2,3-dioxygenase (IDO) activity, an enzyme that catalyzes the breakdown of Trp into Kyn (Kyn/Trp ratio) and is induced by IFN-γ, has been implicated in promoting systemic inflammation and immune dysregulation by increasing Trp catabolism. This study also shows the effects of DAA treatment on IDO activity, recognized as an important immune regulatory enzyme induced in response to chronic viral infections and, in the context of HIV and HCV infection, which represents a marker of disease progression linked to T-cell activation and inflammation levels [21,22,23].

In HCV/HIV co-infected, a decrease in the Kyn/Trp ratio, associated with DAA treatment, is caused by an increase in Trp concentration and a decrease in Kyn concentration in the serum. This is consistent with a decrease in the IDO activation by virally suppressive DAA treatment. Conversely, in HCV mono-infected, higher Kyn, despite unchanged Trp levels, resulted in unmodified IDO activity due to therapy. IDO activity can exert divergent effects in immunosuppression or immune stimulation in numerous conditions, and Kyn metabolism seems to constitute a delicate balance between pathogen defense and host protection [39]. IDO generates a series of metabolites along the Kyn pathway, reported to be upregulated in response to inflammation, and that have suppressive effects on inflammation and immune responses [39]. It is possible that in HCV mono-infected patients, an unbalance of the Kyn pathway downstream of the Trp may occur by the different pathways of inflammation during treated HCV disease.

IDO is an important regulator component in the control of lymphocyte proliferation and in the activation of the type 1 T helper immune response. In contrast, the type 2 cytokines IL-4 and IL-10 inhibit IFN-γ-induced and IDO-mediated Trp catabolism [40]. In this study, expression of IFN-γ showed a similar increasing trend in HCV mono-infected and in HCV/HIV co-infected, which was significantly higher at the end of follow-up, with no statistically significant association between IDO activity and inflammatory activity. It is possible that a type 2 cytokine analysis may contribute to explaining the different responses of IDO activity in HCV mono-infected and HCV/HIV co-infected following DAA treatment.

The small-scale application of our study protocol was intended to verify if the design was appropriate, to establish its feasibility, or derive information to determine the sample size of a larger study. The results from this study provide a preliminary step in our understanding of viral-host interactions immediately after HCV elimination but, more importantly, provide long-term post-SVR follow-up outcomes.

## 5. Conclusions

In summary, considering the overall impact of DAA therapy on the two groups of patients, which are however limited in number, the most relevant evidence was observed in HCV/HIV co-infected patients, with significant effects on the innate immune response, immune phenotype of CD4+ and CD8+ T cells and immune activation that may persist for a long time after the end of treatment. In addition, in HCV mono-infected, T-cell immune responses and the state of immune activation remained elevated for a long time after the end of treatment. As already stated, a limitation of our study includes the relatively small sample size in each study group. Moreover, abandonment of the study during follow-up or lack of compliance by patients did not compromise the results obtained since these patients were similar to the others that completed the study for all the main parameters. However, this study differs from previous works in the comparison between HCV mono-infected and HCV/HIV co-infected patients, for the choice to analyze short and, most importantly, long-term outcomes of DAA treatment, and for the diversity of the selected biomarkers employed to clarify the relative contributions of the innate and adaptive immune system to the pathogenesis of HCV.

This study provides a preliminary step to carry out long-term outcomes that incorporate even more selected biomarkers of a large number of DAA-treated patients to clarify the relative contributions of the innate and adaptive immune system to the pathogenesis of HCV diseases. In fact, the persistence of immune activation could contribute to post-SVR consequences such as progression of liver fibrosis, the risk of reinfection, and hepatocellular carcinoma development. Restoration of immune homeostasis has several potential clinical implications, such as protection from reinfection, from reactivation of other viruses, hepatocellular carcinoma development, and possibly reversal of liver fibrosis. Further, as the normalization of some biomarkers is not achieved, in spite of HCV eradication with DAAs, our data prompt anti-HCV treatment in HCV/HIV co-infected patients at the earliest stages of liver damage to enhance the normalization of systemic inflammation markers.

## Figures and Tables

**Table 1:** Study population characteristics.

	HCV (*n* = 10)	HCV/HIV-1 (*n* = 10)
Age ^a^	52.5 (48–66)	50.5 (48–60)
Sex (male/female) % male	50%	90%
HCV-RNA (copies × 10^6^/mL) (T0) ^a^	2.39 (0.069–14.1)	3.3 (0.39–10.5)
HIV-RNA (copies × 10^6^/mL) (T0)	ND	<37
ALT level, IU/L ^a^	T0 [65.6 (39–154)] T4 [18.5 (13–23)] (*p* = 0.0020) ^†^	T0 [83 (25–383)] T4 [20 (8–45)] (*p* = 0.0059) ^†^
AST level, IU/L ^a^	T0 [48 (34–117)] T4 [16 (9–22)] (*p* = 0.0020) ^†^	T0 [68.5 (16–221)] T4 [20 (15–65)] (*p* = 0.0195) ^†^
GGT level, IU/L ^a^	T0 [67 (16–442)] T4 [19 (11–48)] (*p* = 0.0039) ^†^	T0 [75 (31–209)] T4 [34.5 (20–119)] (*p* = 0.1055) ^‡^
Liver stiffness (kPa) ^a^	T0 [10.1 (4.6–12.5)] T4 [5.4 (2.7–9.9)] (*p* = 0.0050) ^†^	T0 [10 (4–14)] T4 [7 (4–10)] (*p* = 0.0130) ^†^
APRI score	T0 [0.7 (0.5–1.1)] T4 [0.2 (0.1–0.3)] (*p* = 0.0020) ^†^	T0 [0.7 (0.5–1.4)] T4 [0.3 (0.2–0.3)] (*p* = 0.0547) ^‡^
FIB-4 index	T0 [1.8 (1.7–2.4)] T4 [1.1 (0.7–1.4)] (*p* = 0.0020) ^†^	T0 [1.9 (1.3–2.3)] T4 [1.3 (1.2–1.5)] (*p* = 0.1641) ^†^
Genotype 1/others (%)	50%	60%
Genotype 2/others (%)	20%	20%
Genotype 3/others (%)	30%	20%
Sofosbuvir + Daclatasvir	5	6
Sofosbuvir + Ledispavir	5	4

^a^ Data are expressed as median (range). ND (not done). ^†^ Statistically significant difference between T0–T4. ^‡^ Not statistically significant difference between T0–T4.

## Data Availability

The data presented in this study are available only in this manuscript.
